# Supplementary material for: Incommensurate grain-boundary atomic structure
Source: Nat Commun. 2023 Dec 5;14:7806. doi: 10.1038/s41467-023-43536-0 (PMC10697943; doi:10.1038/s41467-023-43536-0)
Supplement: Supplementary file 1 — Supplementary Information [file 41467_2023_43536_MOESM1_ESM.pdf]

Supplementary Information

Incommensurate grain-boundary atomic structure

Takehito Seki[1,2*], Toshihiro Futazuka[1], Nobusato Morishige[3], Ryo Matsubara[4], Yuichi Ikuhara[1,5] & Naoya Shibata[1,5,6*]

[1]*Institute of Engineering Innovation, School of Engineering, The University of Tokyo, Yayoi 2-11-16, Bunkyo-ku, Tokyo 113-8656, Japan.*

[2]*PRESTO, Japan Science and Technology Agency, Kawaguchi, Saitama 332-0012, Japan*

[3]*Kyushu R&D Laboratory, Nippon Steel Corporation, 1-1 Tobihatacho, Tobata-ku, Kitakyushu-shi, Fukuoka 804-8501, Japan.*

[4]*Steel Research Laboratories, Nippon Steel Corporation, 20-1 Shintomi, Futtsu-shi, Chiba 293-8511, Japan.*

[5]*Nanostructures Research Laboratory, Japan Fine Ceramic Center, 2-4-1 Mutsuno, Atsuta-ku, Nagoya 456-8587, Japan.*

[6]*Quantum-Phase Electronics Center (QPEC), The University of Tokyo, Hongo 7-3-1, Bunkyo-ku, Tokyo 113-8656, Japan.*

*Corresponding authors: seki@sigma.t.u-tokyo.ac.jp, shibata@sigma.t.u-tokyo.ac.jp

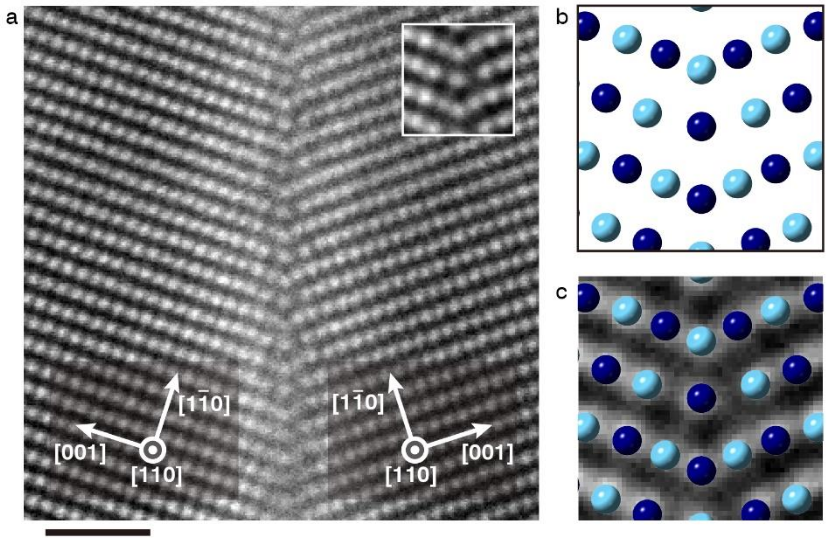

Supplementary Fig. 1: ADF STEM image and structure model of the Σ9 {114} symmetric tilt GB of the Fe-3mass%Si bicrystal. a, ADF STEM image. The inset is the averaged image of the GB structure units. The scale bar represents 1 nm. **b,** The most stable GB structure derived from the simulated annealing. **c,** The averaged ADF image of the GB structure units superimposed with the structure model.

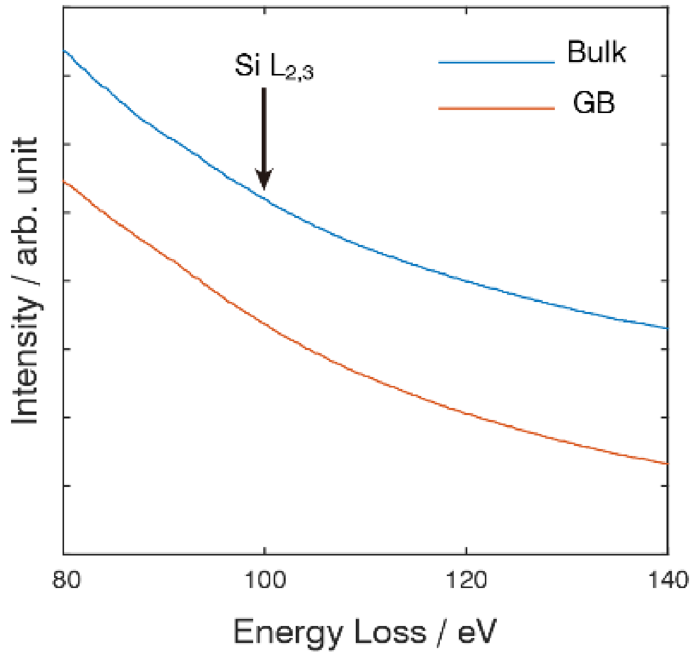

Supplementary Fig. 2: EEL spectra obtained from bulk and GB regions. These spectra were obtained by scanning the electron probe in the regions approximately 3 nm away from the grain boundary and within approximately 1 nm regions on the grain boundary core, respectively.

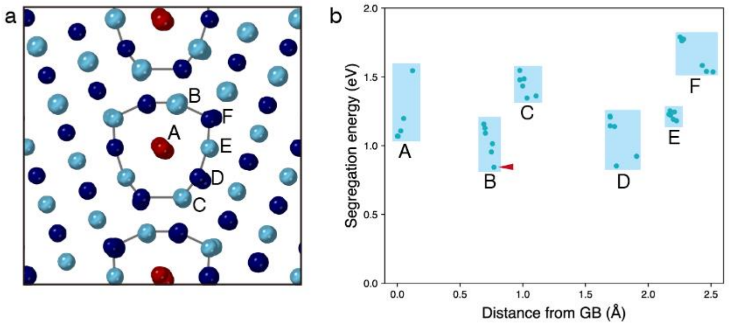

Supplementary Fig. 3: The segregation energies of Si at Σ9 {221} GB. a, The atomic structure of Σ9 {221} GB. The black triangle indicates the center of GB. The Fe atoms at the center and two different layers along the [110] viewing direction are colored red, blue, and light blue, respectively. **b**, The segregation energies of substitutional Si in Fe sites as a function of distance from the center of the GB. The atoms in the same atomic columns are surrounded by blue rectangles. The red arrow corresponds to the most stable segregation site.

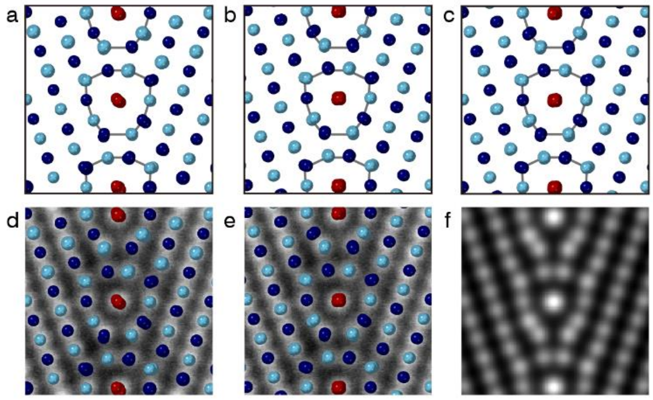

Supplementary Fig. 4: Structure models predicted by MD calculations and the simulated STEM image. a,b,c The most stable GB structures predicted with 1 × 3, 1 × 5 and 1 × 8 simulation cells, respectively. **d,e,** The experimental ADF STEM images superimposed with the structure models shown in **a** and **b**, respectively. **f**, The simulated ADF STEM image using the structure model shown in **c**.

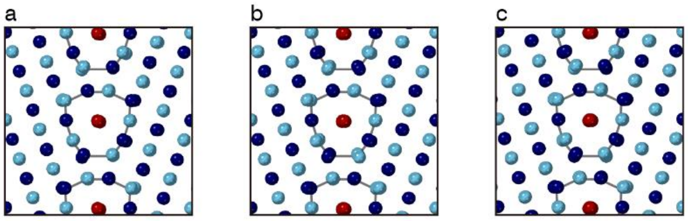

Supplementary Fig. 5: Structure models predicted by DFT calculations. a,b,c, The stable structure models predicted by the 1×3, 1×5, and 1×8 cells, respectively. Here, the most stable structures derived by the simulated annealing shown in Fig. 2**b** and Supplementary Fig. 4 were further relaxed by DFT calculations.

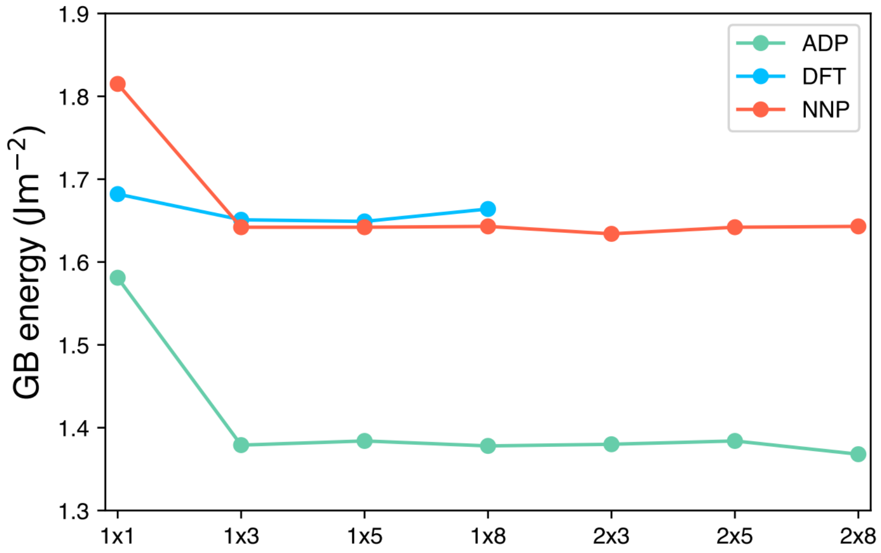

Supplementary Fig. 6: GB energies of the most stable GB structures for each cell size derived by MD (ADP, NNP) and DFT. It is found that the basic trend of energy vs. cell size and the obtained GB atomic structures are the same in the results of ADP and NNP. However, DFT results show some discrepancy. For DFT calculations, we assumed ferromagnetic spin configuration as the initial structures before structural relaxation. Here, we cannot exclude the possibility that the obtained GB energies are trapped in the metastable spin configurations by the present DFT calculations, as demonstrated in Supplementary Fig. 13. On the other hand, NNP has more flexible function form than ADP, and is a well-tuned NNP achieves higher accuracy than conventional interatomic potentials such as ADP. Although we cannot directly specify the spin states of the system, the spin is indirectly incorporated into the potential by fitting to the results of DFT

calculations with various spin states. Therefore, NNP can imitate DFT calculation including the effect of spin configurations and find the global energy minimum structures. The absolute energy values become comparable to the DFT results, showing better energy accuracy.

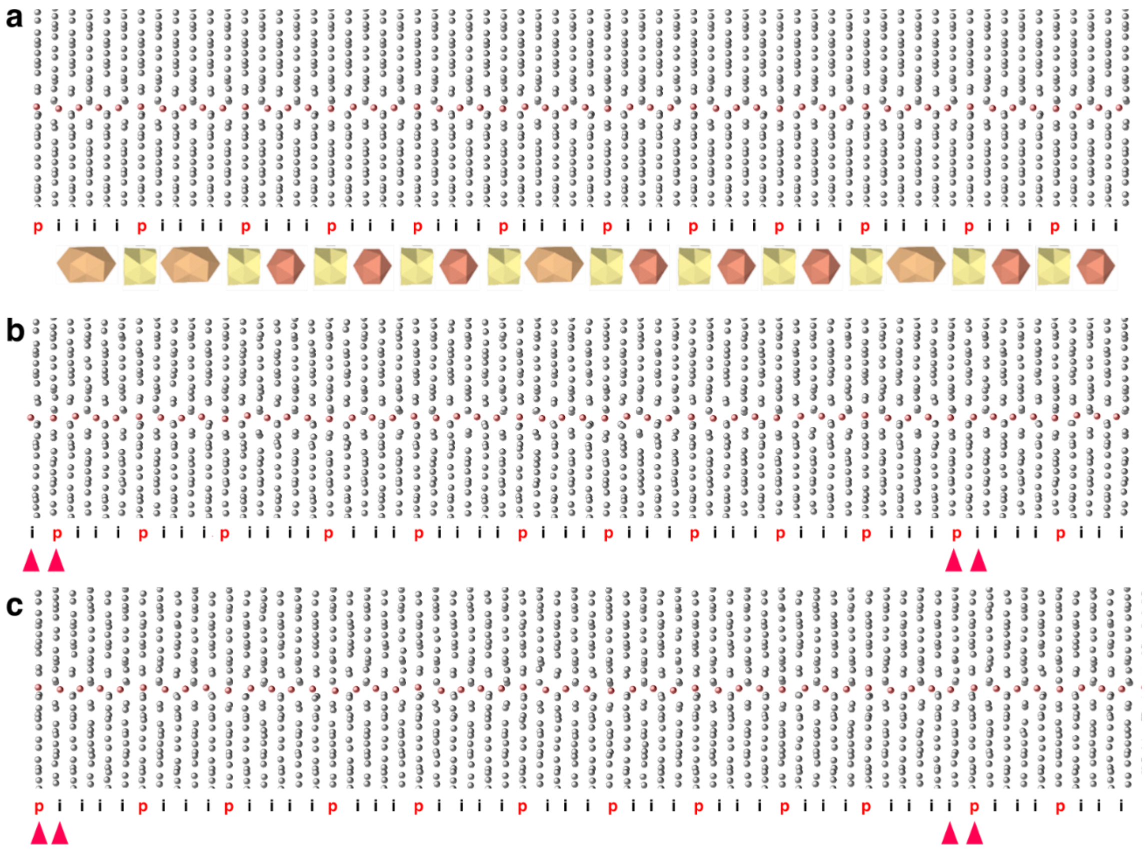

Supplementary Fig. 7: The atomic structure of the long-period 1×32 cell GB. a, The stable structure of the 1×32 cell GB simulated with ADP potential. The core atoms are shown with red color and the labels i and p indicates the interlayer site and the center of pentagons, respectively. **b** and **c** are snapshots from constant temperature MD simulation at 14.50 ps and 15.00 ps, respectively. The core Fe atoms frequently moved during the simulation, and the GB atomic structures fluctuated between inequivalent structures from moment to moment. The red arrow indicates the sites at which large atomic fluctuations are observed.

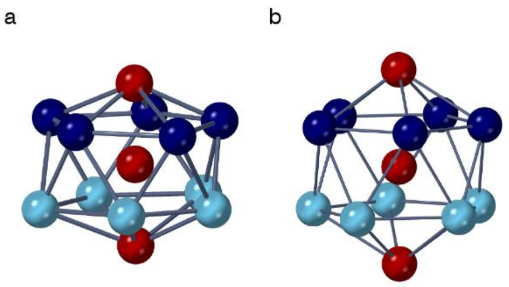

Supplementary Fig. 8: Structure model of icosahedral clusters. a, Hypothetical icosahedral cluster formed with the same periodicity as the surrounding bulk crystal environment. **b**, Ideal icosahedral cluster.

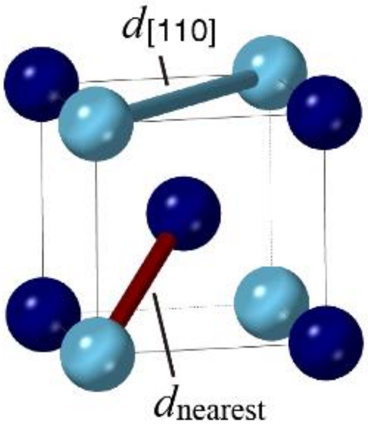

Supplementary Fig. 9: Structure model representing the atomic distances in the body-centered cubic structure. $d_{[110]} = \sqrt{2}a$, $d_{\mathrm{nearest}} = \sqrt{3}a/2$ (a denoting the lattice constant.) The distance ratio $d_{[110]}/d_{\mathrm{nearest}}$ is calculated to be $2\sqrt{6}/3 \simeq 1.625$, which is equal to the relative density of the central atomic column with reference to that of the atomic columns in the bulk under the assumption that the interatomic distance of the central atoms is d_{nearest}.

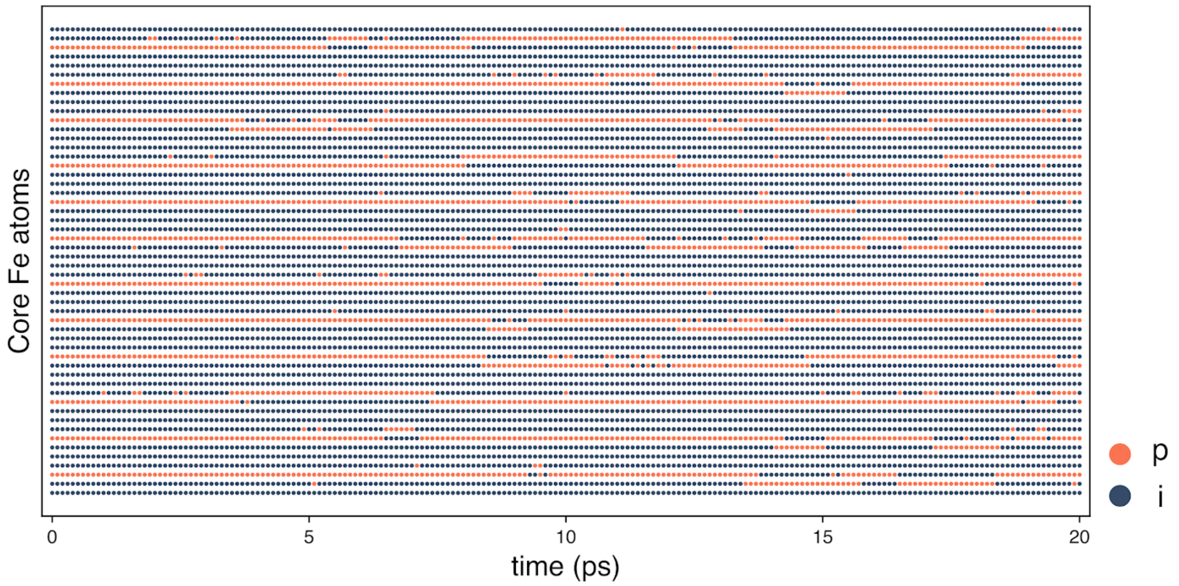

Supplementary Fig. 10: Time evolution of the core atom positions in 1×32 cell GB at 200 K. From the trajectory of MD simulation, the core atom positions are classified as p (pentagonal) or i (interlayer), which are shown as red and blue filled circles, respectively. The core atoms are classified as p if their projected positions are within 0.3 Å of the bulk projected positions. As the time evolves, some of the core atoms transit to different positions, making the overall GB atomic structure different before and after the transition.

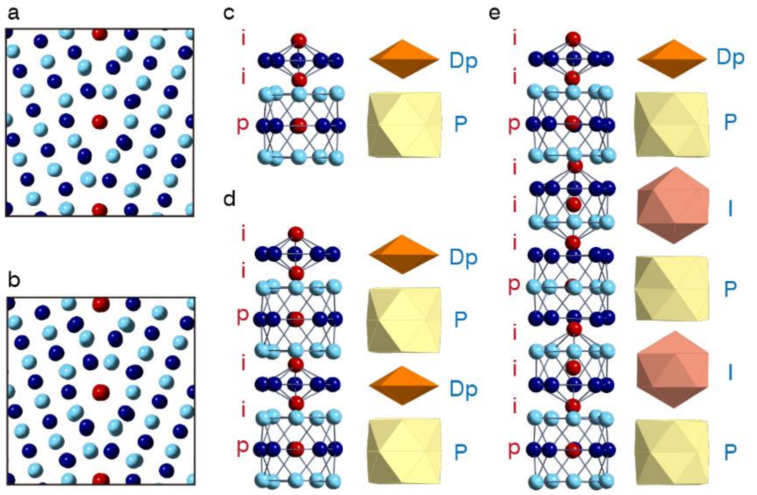

Supplementary Fig. 11: The stable GB atomic structures of 1×2, 1×4, and 1×7 cells.

a, **b** show the atomic structures of 1×2 (1×4) and 1×7 GBs observed along the [110] axis. Note that the 1×2 and 1×4 GBs have the same atomic structure. The GB atomic structures viewed along the [110] axis are similar to those of 1×3, 1×5, 1×6, 1×8 cells. **c**, **d**, **e** show the atomic structures of 1×2, 1×4, and 1×7 GBs observed along the orthogonal direction. In these GBs, in addition to the icosahedral cluster (I) and the double-pentagonal antiprism (P), the pentagonal dipyramid clusters (Dp) are formed in the GB core.

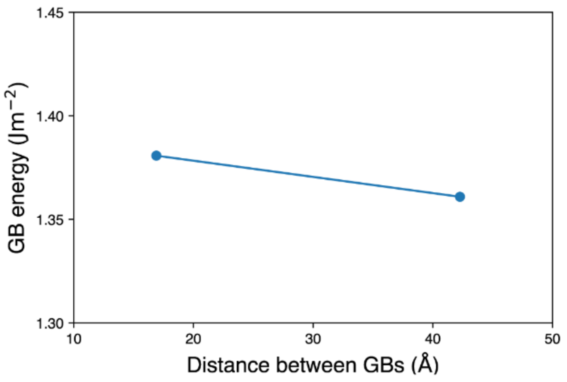

Supplementary Fig. 12: The relationship between the GB separation distance in the simulation cell and the GB energy of Σ9 {221} (1×3) GB. GB energies were calculated with the GB separation distances of 16.90 and 42.27 Å.

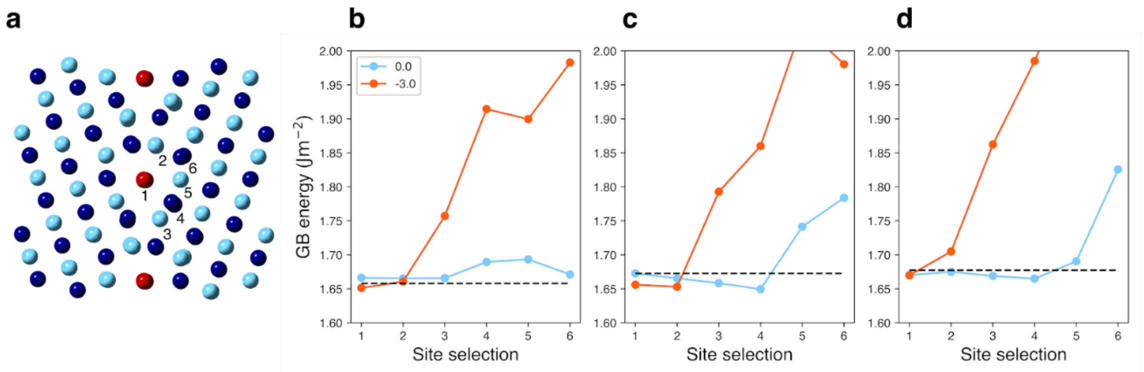

Supplementary Fig. 13: The GB energies calculated from the different initial magnetic moments and configurations by DFT. The initial magnetic moments of the Fe atoms as indicated by sites from 1 to 6 in **a** are gradually changed to be 0.0 or -3.0, while keeping the other atoms' magnetic moment to be 3.0. **b**, **c**, and **d** show the GB energies of 1×3, 1×5, and 1×8 GB, respectively, calculated from the different initial magnetic moments and configurations. The blue and orange lines correspond to the initial magnetic moments of 0.0 and -3.0, respectively. The dashed lines show the GB energy calculated from the ferromagnetic magnetic configurations as shown in Supplementary Fig. 6, where the initial magnetic moments are set to be 3.0 for all the Fe atoms.

Cell size	ADP (J/m^2)	DFT (J/m^2)	NNP (J/m^2)
1×1	1.581	1.682	1.815
1×3	1.379	1.651	1.642
1×5	1.384	1.649	1.642
1×8	1.378	1.664	1.643
2×3	1.380	-	1.634
2×5	1.384	-	1.642
2×8	1.368	-	1.643

Supplementary Table 1: GB energies of the stable GB structures for each cell size derived from MD (ADP and NNP) and DFT calculations.
