# Supplementary material for: Incommensurate grain-boundary atomic structure
Source: Nat Commun. 2023 Dec 5;14:7806. doi: 10.1038/s41467-023-43536-0 (PMC10697943; doi:10.1038/s41467-023-43536-0)
Supplement: Supplementary file 3 — Description of Additional Supplementary Files [file 41467_2023_43536_MOESM3_ESM.pdf]

Description of Additional Supplementary Files

Supplementary Movie 1: Movie of structural fluctuations of the 1×8 grain boundary structure.
